# Late-Onset Exudative Pleural Effusions Without Concomitant Airway Obstruction or Lung Parenchymal Abnormalities: A Novel Presentation of Chronic Lung Allograft Dysfunction

**DOI:** 10.3389/ti.2024.12395

**Published:** 2024-01-26

**Authors:** Devika Sindu, Sandhya Bansal, Bhuvin Buddhdev, Kendra McAnally, Hesham Mohamed, Rajat Walia, Thalachallour Mohanakumar, Sofya Tokman

**Affiliations:** ^1^ Norton Thoracic Institute, Phoenix, AZ, United States; ^2^ School of Medicine, Creighton University, Phoenix, AZ, United States

**Keywords:** chronic lung allograft dysfunction, exudative pleural effusion, lung transplant, small extracellular vesicles, new CLAD phenotype

## Abstract

Restrictive allograft syndrome (RAS) is an aggressive variant of CLAD characterized by progressive restrictive ventilatory decline and persistent pleuro-parenchymal changes that can be seen on chest CT. We identified four lung transplant recipients with a progressive restrictive ventilatory defect due to lymphocyte-predominant exudative pleural effusions, but no pleuro-parenchymal abnormalities typical of RAS. Using molecular analysis, we also found increased levels of previously described immune markers of RAS, including NFkB, 20S proteasome, lipocalin, TNFα, and TGFβ, within the circulating small extracellular vesicles of the remaining living lung transplant recipient. Despite the absence of lung parenchymal changes, these patients had a poor prognosis with rapid deterioration in allograft function and no response to pleural-based interventions such as thoracentesis, decortication, and pleurodesis. We hypothesize that these cases represent a distinct CLAD phenotype characterized by progressive restriction due to pleural inflammation, lymphocyte-predominant pleural effusion, resultant compressive atelectasis, and eventual respiratory failure in the absence of lung parenchymal involvement.

## Graphical Abstract

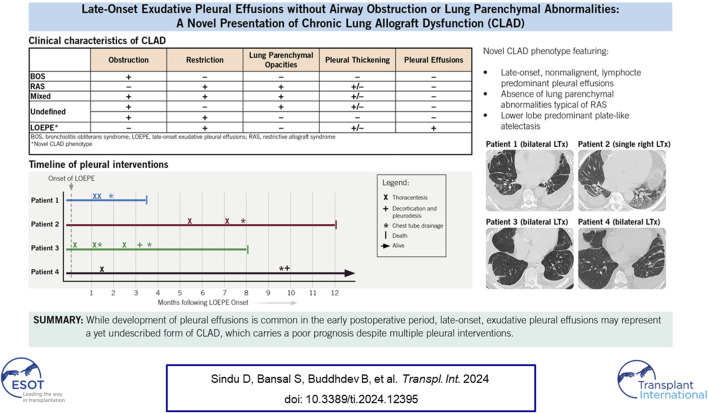

## Introduction

Chronic lung allograft dysfunction (CLAD) remains a major challenge after lung transplantation (LT), limiting long-term survival and graft function in lung transplant recipients (LTRs). Bronchiolitis obliterans syndrome (BOS) is the most common phenotype of CLAD, seen in 50%–70% of cases, and is characterized by progressive, irreversible airflow obstruction due to bronchiolar inflammation and fibroproliferation [1]. Restrictive allograft syndrome (RAS), a less prevalent but distinct phenotype, is seen in 10%–30% of LTRs with CLAD and is characterized by restrictive spirometry changes, persistent radiographic opacities, and a markedly worse prognosis [2–4]. The 2019 consensus report from the Pulmonary Council of the International Society of Heart and Lung Transplantation also describes mixed and undefined phenotypes to characterize combinations of CLAD presentations [5]. Studies have shown that manifestations of CLAD are heterogeneous, and its diagnosis requires a methodical, guideline-based approach to identify novel phenotypes [2, 5–10]. In addition to clinical phenotypic differences, translational studies indicate that BOS and RAS may also have distinct immunologic profiles [11]. In this case series, we describe four LTRs with a restrictive ventilatory defect characterized by the development of exudative pleural effusions, pleural thickening, and plate-like or rounded atelectasis. We hypothesize that these cases represent a distinct CLAD phenotype with a poor prognosis (Table 1).

**TABLE 1:** Baseline characteristics and clinical features of lung function impairment.

	Patient 1	Patient 2	Patient 3	Patient 4
Baseline clinical characteristics				
Sex	Male	Male	Male	Male
Age at LT, years	64	72	67	63
Indication for LT	IPF	HP	IPF	IPF
Type of LT (single vs. double)	Double	Single, right	Double	Double
PGD-3 at 72 h	No	No	No	No
CMV serostatus (D/R)	D+/R-	D+/R+	D+/R-	D+/R+
Clinical characteristics at CLAD onset^a^				
Age at CLAD onset	69	72.5	69.5	65
Immunosuppressive regimen	Tac, MMF, CS	Tac, MMF, CS	Tac, MMF, CS	Tac, MMF, CS
DSA	negative	negative	negative	negative
Transthoracic echocardiogram				
Systolic function (LVEF)	50%–60%	60%–65%	60%–65%	60%–65%
Diastolic dysfunction	none	Grade 1, mild	Grade 1, mild	Grade 2, moderate
Right heart catheterization				
Mean PAP	26 mm Hg	22 mm Hg	14 mm Hg	18 mm Hg
PCWP	16 mm Hg	15 mm Hg	6 mm Hg	9 mm Hg
Imaging after CLAD onset				
Chest CT	Bilateral pleural effusions, pleural thickening, plate-like and rounded atelectasis	Right-sided pleural effusion, pleural thickening, plate-like and rounded atelectasis	Bilateral pleural effusions, pleural thickening, plate-like and rounded atelectasis	Bilateral pleural effusions, pleural thickening, plate-like and rounded atelectasis
Lung allograft parenchymal changes typical of RAS, consolidations, ground glass opacities, or fibrosis	No	No	No	No
Bronchoscopic findings after CLAD onset				
BAL cultures	No growth	No growth	No growth	No growth
Transbronchial biopsy	A0B0	A0B0	A0B0	A0B0
Pleural effusion: characteristics and management				
Pleural fluid characteristics at CLAD onset^a^	Exudative	Exudative	Exudative	Exudative
White cell count (lymphocytes %)	197/uL (72%)	4083/uL (92%)	102/uL (65%)	2637/uL (87%)
LDH	241 U/L	268 U/L	242 U/L	233 U/L
Protein	2.2 g/dL	3.8 g/dL	4.2 g/dL	4.4 g/dL
Management of pleural effusion	thoracentesis, chest tube drainage	thoracentesis, chest tube drainage	thoracentesis, chest tube drainage, decortication, pleurodesis	thoracentesis, chest tube drainage, decortication, pleurodesis
Outcomes and time measures				
Deceased	Yes	Yes	Yes	No
Cause of death	respiratory failure	respiratory failure	respiratory failure	—
Time from LT to initial FEV_1_ decline, months	62	6	30	20
Time from initial FEV_1_ decline to death, months	3.5	12	8	—
Time from LT to death, months	65.5	18	38	—

^a^ CLAD, onset is marked by ≥20% decline in FEV_1_ for >3 months. The initial FEV_1_ decline also coincides with the new onset recurrent pleural effusions.Abbreviations: BAL, bronchoalveolar lavage; BOS, bronchiolitis obliterans; CLAD, chronic lung allograft dysfunction; CMV, cytomegalovirus; CS, corticosteroids; D/R, donor/recipient serostatus; DSA, donor- specific antibodies; FEV_1_, forced expiratory volume in 1 s; FVC, forced vital capacity; HLA, human leukocyte antigen; HP, hypersensitivity pneumonitis; IPF, idiopathic pulmonary fibrosis; LDH, lactate dehydrogenase; LT, lung transplant; LVEF, left ventricular ejection fraction; MMF, mycophenolate mofetil; PAP, pulmonary artery pressure; PCWP, pulmonary capillary wedge pressure; PFT, pulmonary function test; PGD, primary graft dysfunction; RAS, restrictive allograft syndrome; Tac, tacrolimus.

## Materials and Methods

This study was approved by our Institutional Review Board (PHX-21-500-198-73-18 dated 07/12/2023) with the need for informed consent waived as data was collected retrospectively by chart review. Analyses of small extracellular vesicles (sEVs) were conducted after approval by our Institutional Review Board (PHXB-16-0027-10-18 dated 7/14/2020) and after obtaining written informed consent from the participant. Small extracellular vesicles were isolated from plasma samples and characterized by Western blot (Supplementary Methods). Student’s *t* test and paired *t*-test were used when appropriate to compare the relative densities of sEVs isolated from the samples. Statistical analyses were carried out using Prism (GraphPad Software). All patient care was carried out under strict compliance with the International Society of Heart and Lung Transplantation ethics statement.

## Results

### Patient 1

**FIGURE 1:**
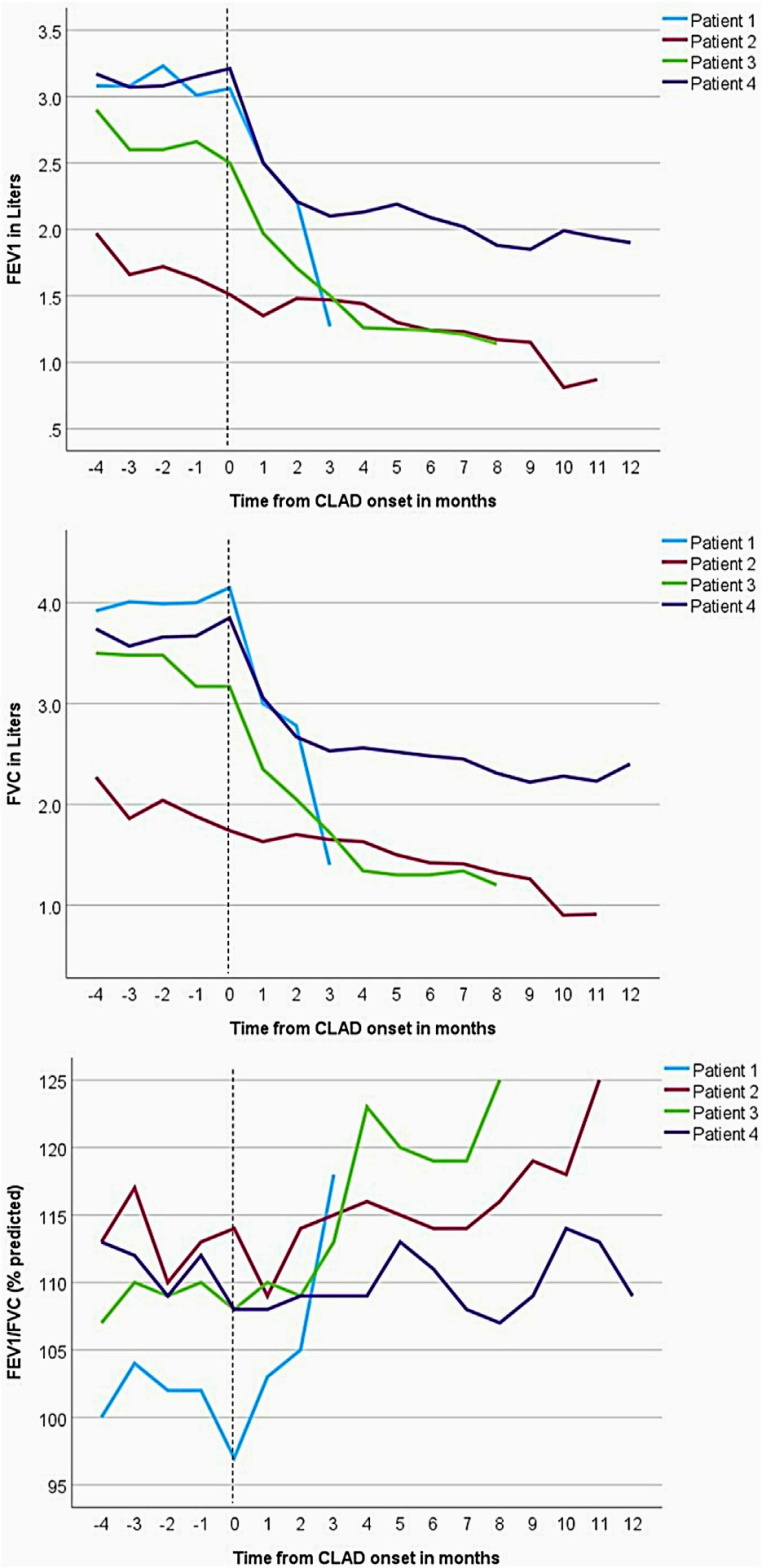
Forced expiratory volume in the first second (FEV_1_), forced vital capacity (FVC), and FEV_1_/FVC trends in the four patients. Spirometry shows a restrictive pattern of decline in lung allograft function. The vertical dashed lines represent the onset of chronic lung allograft dysfunction (CLAD), marked by recurrent pleural effusions.

**FIGURE 2:**
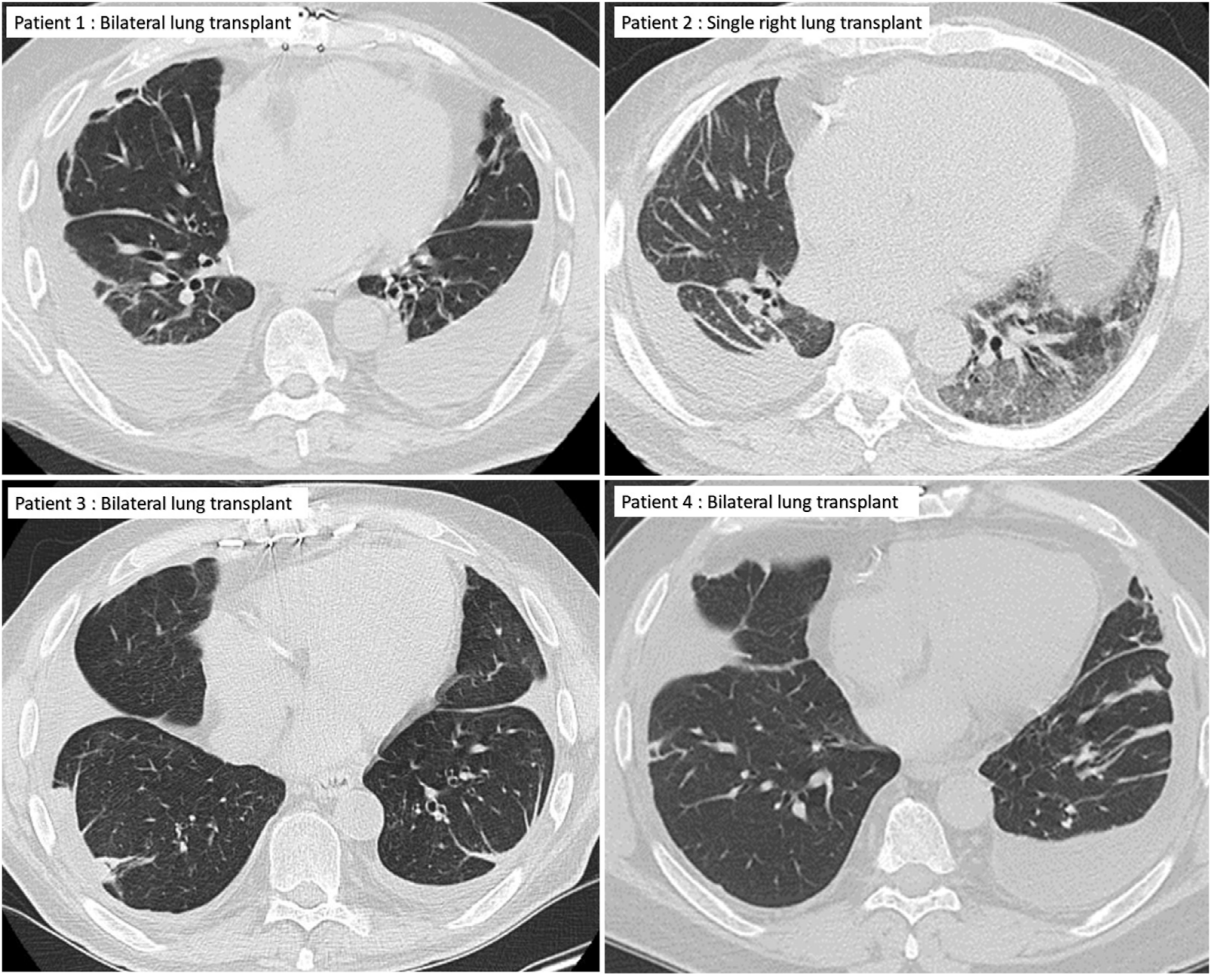
Chest CT of the four patients showing pleural effusions and plate-like atelectasis but no lung parenchymal changes typical of restrictive allograft syndrome.

A 64-year-old man with idiopathic pulmonary fibrosis underwent bilateral LT. His post-LT course was complicated by an episode of CMV viremia, bilateral pleural effusions, and bronchomalacia requiring left main stem bronchus stent placement. He subsequently enjoyed an active lifestyle with stable allograft function and was maintained on standard 3-drug immunosuppression with tacrolimus, mycophenolate mofetil (MMF), and prednisone. Three years after transplant, he developed recurrent squamous cell skin cancer, and MMF was transiently replaced with everolimus in an attempt to slow cancer progression and recurrence. One year later, he had a 15% drop in FEV_1_, was treated with an empiric 3-day course of 500 mg methylprednisolone, transitioned off of everolimus and back on MMF, and underwent bronchoscopy with stent placement for severe left bronchomalacia. His lung function returned to baseline and remained stable for the next 9 months but began to precipitously drop thereafter, with an FEV_1_ decline from 3.06 L (87% predicted) to 2.22 L (63%) in 2 months, and then to a nadir of 1.37 L (39% predicted) in another 1.5 months (Figure 1). His initial evaluation revealed bilateral costophrenic blunting on chest X-ray, prompting a left-sided thoracentesis with withdrawal of 650 mL of culture-negative, lymphocyte predominant (72%), and exudative pleural fluid. The effusion re-accumulated within a week, and a subsequent high-resolution chest CT showed pleural thickening, bilateral pleural effusions, pericardial effusion, and plate-like and rounded atelectasis (Figure 2). Notably, his lung parenchyma appeared normal. Bronchoscopy with bronchoalveolar lavage (BAL) and transbronchial biopsies (TBBx) showed no evidence of infection or acute cellular rejection (ACR); he also never developed donor-specific antibodies (DSAs). He underwent chest tube placement with drainage of pleural fluid and multiple subsequent thoracenteses, which did not improve spirometric flows. A transthoracic echocardiogram showed preserved left ventricular function (55%–60%) and normal diastolic filling. Although a cardiac MRI showed anterior pericardial thickening, functional findings did not meet the criteria for constrictive pericarditis. He died from progressive respiratory failure 64 months after bilateral LT, 3.5 months after his initial drop in FEV_1_.

### Patient 2

A 72-year-old man with a history of hypersensitivity pneumonitis, coronary artery bypass grafting, and gastroesophageal reflux treated with esophageal fundoplication underwent a single right LT. He was maintained on standard 3-drug immunosuppression. His FEV_1_ peaked 1 month after lung transplant [1.97 L (66%)] but began to progressively decline at 6 months, reaching a nadir of 0.87 L (29%) 16 months after LT (Figure 1). The decline in spirometric flows was accompanied by a unilateral, right pleural effusion (Table 1). A subsequent chest CT revealed right-sided pleural thickening, plate-like and rounded atelectasis, and a notable absence of RAS-like parenchymal changes within the allograft (Figure 2). Bronchoscopy with BAL and TBBx showed no evidence of infection or ACR, and he never developed DSAs. He had mild esophageal dysfunction characterized by esophageal stasis and motor incoordination on esophagram but showed normal manometric findings and no evidence of gastroesophageal reflux with a DeMeester score of 1.1. A transthoracic echocardiogram showed normal left ventricular ejection fraction (60%–65%) with mild left ventricular diastolic dysfunction and a raised right ventricular systolic pressure (42 mm Hg). He had a follow-up right heart catheterization (RHC), which revealed a pulmonary arterial pressure (PAP) of 39/11 mm Hg (mean 22 mm Hg) and a pulmonary capillary wedge pressure (PCWP) of 15 mm Hg. Multiple thoracenteses and chest tube drainage did not improve spirometric flows, and analysis revealed culture-negative, exudative, lymphocyte-predominant (92%) pleural fluid. Decortication and pleurodesis were deferred as he was deemed high-risk due to his inability to tolerate single left lung ventilation. He died of respiratory failure 17 months after LT, 12 months after his initial drop in FEV_1_.

### Patient 3

A 67-year-old man with idiopathic pulmonary fibrosis underwent bilateral LT and was maintained on standard 3-drug immunosuppression. His posttransplant gastroesophageal evaluation revealed reflux with an elevated DeMeester score of 45.1 and poor peristalsis on manometry. He was offered fundoplication, but the patient elected medical management with aspiration precautions. He was hospitalized at an outside institution with worsening dyspnea and new bilateral pleural effusions 30 months after LT. His transthoracic echocardiogram revealed normal left ventricular systolic function and mild diastolic dysfunction. Bronchoscopy with BAL and TBBx showed no evidence of infection or ACR. Aspiration was thought to contribute to his respiratory decline, and he was transitioned from an oral diet to a trial of tube feeding but continued to have a decline in spirometric flows (Figure 1) and recurrent pleural effusions (Figure 2) with a lack of appropriate lung parenchymal expansion after thoracentesis or chest tube placement. Pleural fluid analysis revealed a lymphocyte-predominant (65%), exudative effusion, and his chest CT showed pleural thickening, plate-like and rounded atelectasis within the allograft, and an absence of RAS-like lung parenchymal changes. He subsequently underwent a partial right decortication and bilateral pleurodesis, but his spirometric flows continued to decline, with his FEV_1_ reaching a nadir of 1.14 L (35% predicted) 7 months after his initial decline. He died 1 month later from respiratory failure, 38 months after LT and 8 months after his initial drop in FEV_1_.

### Patient 4

**FIGURE 3:**
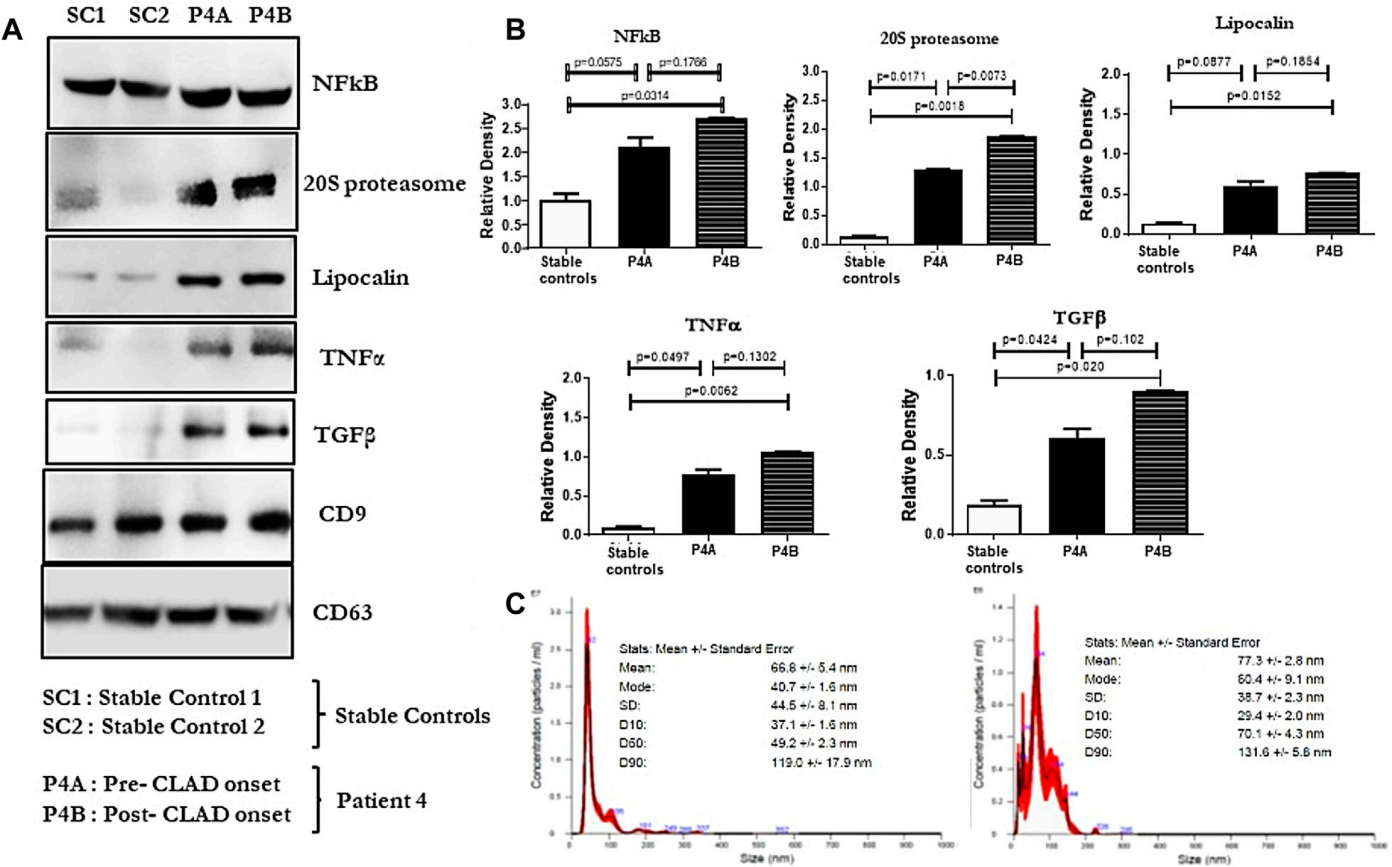
**(A)**: Western blot of small extracellular vesicle (sEV) proteins NFkB, 20S proteasome, lipocalin, TNFα, and TGFβ from plasma samples of lung transplant recipients. SC1 and SC2 denote sEV proteins from 2 stable controls; P4A and P4B denote sEV proteins in the plasma of patient 4, 9 months before and 1 year after the onset of CLAD, respectively. CD9 and CD63 were used as controls to normalize the blots for NFkB, 20S proteasome, lipocalin, TNFα, and TGFβ. **(B)**: Densitometry and statistical analysis of western blots for stable controls and pre and post CLAD-onset samples of patient 4. Relative density graphs are represented as bar plots. **(C)**: Nanosight images depicting the size of the sEVs isolated from both samples of patient 4.

A 63-year-old man with idiopathic pulmonary fibrosis underwent bilateral LT and was maintained on standard 3-drug immunosuppression. He had an uneventful posttransplant course until he developed a gradual spirometric decline 20 months after transplant (Figure 1). His chest CT initially showed bilateral, loculated pleural effusions, and subsequent imaging revealed pleural thickening and plate-like and rounded atelectasis but no RAS-like lung parenchymal changes (Figure 2). Bronchoscopy with BAL and TBBx showed no evidence of infection or ACR, and he never developed DSAs. A transthoracic echocardiogram showed normal left ventricular systolic function but moderate left ventricular diastolic dysfunction. He also underwent RHC, which showed no evidence of pulmonary arterial hypertension [PAP: 28/9 mm Hg (mean 18 mm Hg); PCWP: 9 mm Hg]. He was on chronic diuretic therapy and had multiple thoracentesis procedures, which did not improve spirometric flows, and analysis revealed culture-negative, exudative, lymphocyte-predominant (87%) pleural fluid. He then underwent video-assisted thoracoscopic surgery with decortication and doxycycline pleurodesis 9 months after his initial spirometric decline. A pleural biopsy (Supplementary Figure S1) showed pleural fibrosis with organizing hemothorax, and he received two doses of intrapleural tissue plasminogen activator to break up loculations. His spirometric flows initially stabilized postoperatively, but he complained of intractable chest pain and dyspnea on exertion. In addition, sEVs isolated from the patient’s plasma contained elevated levels of NFkB, 20S proteasome, lipocalin, TNFα, and TGFβ compared to control samples from stable LTRs (Figure 3; Supplementary Figure S2), and the concentrations of these inflammatory immunologic markers increased before the onset of CLAD. He remains alive 33.5 months after LT; however, his FEV_1_ is at a nadir of 1.9 L, a 39.7% decline from baseline.

## Discussion

Chronic lung allograft dysfunction is the most common cause of death among long-term survivors of LT. In 2019, Verleden et al [5] published a consensus statement defining and standardizing the nomenclature and clinical phenotypes of CLAD to facilitate collaboration between centers investigating its pathogenesis, prevention, and treatment. They defined CLAD as a substantial and persistent decline (≥20%) in FEV_1_ from baseline, with a predominantly obstructive, predominantly restrictive, or mixed obstructive and restrictive ventilatory pattern that is not explained by other conditions, including pleural effusion. A second consensus statement in the same year by Glanville et al [2] specifically focused on RAS and defined its diagnostic criteria as a ≥20% decline in FEV_1_ from baseline and persistent opacities on chest imaging. The absence of RAS-like radiographic opacities [4, 8] and the presence of pleural effusions among our patients excludes them from the currently accepted definitions of RAS, thereby highlighting their unique physiology and phenotype.

Development of recurrent pleural effusion is a well-known LT complication [12, 13], but a yet undescribed manifestation of CLAD. In a large German study of 1223 LTRs, Joean et al [14] identified 113 (9.2%) patients with clinically significant pleural effusions requiring thoracentesis. They observed a bimodal distribution of pleural effusion onset with 67 (59%) patients developing pleural effusion within the first 6 months after LT at a median of 63 days [interquartile range (IQR) 39–96 days] followed by a second peak in 46 patients (41%) who developed pleural effusion at a median of 838 days (IQR 287–1,197 days). The odds of developing a malignant effusion or a cardiogenic effusion were significantly higher in the late-onset group [OR 3.55; CI (1.11–11.32) and OR 5.96; 95% CI (1.95–18.17), respectively], and the late-onset group had lower overall survival than a matched control group [HR 2.43, 95% CI (1.27, 4.62), *p* < 0.05]. However, the survival difference did not retain statistical significance after excluding malignant pleural effusions. In contrast, the LTRs in our series showed a markedly reduced survival rate and poor prognosis after the development of late-onset, exudative, nonmalignant pleural effusions, despite the absence of a concurrent illness to drive morbidity and mortality. This supports our hypothesis that the pleural effusions seen in our cohort are different from previously described late-onset pleural effusions [12] and instead represent a phenotypically unique manifestation of CLAD.

Consequent to persistent and treatment-refractory pleural effusions, the LTRs in our case series developed a progressive restrictive ventilatory defect with spirometric declines mirroring those of RAS (>20% decline in FEV_1_, concurrent FVC decline, FEV_1_/FVC >0.7 in all four patients along with >10% TLC decline in one patient). However, an important phenotypic distinction remains between our patients and patients with RAS—our patients never developed pulmonary opacities typical of RAS. The radiographic patterns of RAS on chest CT typically include parenchymal abnormalities followed by progressive pleuro-pulmonary fibrosis [2, 4, 8, 15, 16]. Dettmer et al [15] developed a CT-score for inflammation based on the presence of central and peripheral consolidations, central and peripheral ground-glass opacities, and pleural abnormalities. Patients with restrictive CLAD had an inflammation score >2 (mean 3.43 vs. 0.60 for patients without restrictive CLAD, *p* < 0.001) and had significantly shorter survival than patients with a score ≤2. In contrast, LTRs in our study had an inflammation score ≤2 due to the absence of lung parenchymal abnormalities, but still developed life-limiting and rapidly progressive respiratory failure. Furthermore, while pleural thickening and fibrosis are well-described abnormalities among patients with RAS, the presence of pleural effusions is unique to this cohort and supports our hypothesis that this may be a novel presentation of CLAD.

Our group has previously demonstrated that elevated levels of immunologic markers can be found in circulating sEVs before the onset of CLAD [17], and the exosomal contents between patients with BOS and those with RAS can vary [11]. Veraar et al [18] also showed that lipocalin-2 was elevated in patients with RAS, and increased serum concentrations predicted worse CLAD-free survival in stable patients. Furthermore, Sacreas et al [19] suggested that the fibrotic process of RAS mirrors that of idiopathic pulmonary fibrosis and may be driven by mesothelial-to-mesenchymal transition, characterized by differentiation of pleural mesothelial cells into myofibroblasts after stimulation by TGFβ. All of these findings align with our preliminary data, which showed elevated levels of NFkB, 20S proteasomes, lipocalin, and TGFβ in circulating sEVs isolated from patient 4 before and after the onset of CLAD. Lastly, Iasella et al [20] demonstrated enhanced type-1 immunity among patients with CLAD, characterized by an increase in the concentration of BAL and airway epithelial inflammatory markers including TNFα. We also detected elevated levels of TNFα in patient 4, but within circulating sEVs rather than the airways. These findings suggest that the immunologic and inflammatory milieu identified in our patient may mirror that of other patients with CLAD, despite the difference in their clinical presentation.

Our study is descriptive in nature and has a small number of patients, thereby limiting our ability to draw definitive conclusions. In addition, TLC was not measured uniformly in all patients, and the death of 3 of 4 patients precluded molecular analysis. However, despite these limitations, our observations remain novel and important, as recognition of specific CLAD phenotypes and a better understanding of CLAD subpopulations are essential for developing novel diagnostic and therapeutic strategies [7, 19, 21, 22]. Currently, patients who do not fit into one of the four main categories of CLAD outlined in the 2019 consensus statement (BOS, RAS, mixed, or undefined) remain unclassified, as is the case with the patients in our cohort, who have a restrictive ventilatory defect, exudative pleural effusions, and no evidence of RAS-like opacities. As Levy et al [7] highlighted in 2020, a classification system that permits a sizable portion of patients to be unclassified will be problematic in future clinical decision-making. Further research is vital to unravel the underlying mechanisms of CLAD [23], refine diagnostic criteria, and develop tailored therapeutic strategies. This is especially important in our patient cohort as multiple interventions including pleural fluid drainage, decortication, and pleurodesis proved morbid and ineffective, despite having a potential therapeutic target within the pleural space. Larger studies are needed to confirm our findings and guide therapeutic interventions in this unusual subset of LTRs.

## Data Availability

The original contributions presented in the study are included in the article/Supplementary Material, further inquiries can be directed to the corresponding author.

## Abbreviations

ACR, acute cellular rejection; BAL, bronchoalveolar lavage; BOS, bronchiolitis obliterans syndrome; CLAD, chronic lung allograft dysfunction; DSA, donor-specific antibodies; FEV_1_, forced expiratory volume in 1 s; LT, lung transplant; LTR, lung transplant recipient; PAP, pulmonary artery pressure; PCWP, pulmonary capillary wedge pressure; RAS, restrictive allograft syndrome; TBBx, transbronchial biopsy.
