# Supplementary material for: Late-Onset Exudative Pleural Effusions Without Concomitant Airway Obstruction or Lung Parenchymal Abnormalities: A Novel Presentation of Chronic Lung Allograft Dysfunction
Source: Transpl Int. 2024 Jan 26;37:12395. doi: 10.3389/ti.2024.12395 (PMC10866027; doi:10.3389/ti.2024.12395)
Supplement: Supplementary file 1 [file DataSheet1.docx]

**SUPPLEMENTAL METHODS**

**Isolation and validation of small extracellular vesicles**

Small extracellular vesicles (sEVs) were isolated from plasma samples using the Invitrogen Exosome Precipitation Kit. The size of the sEVs was determined by NanoSight (Malveran NS300).

**Characterization of sEVs using western blot**

Total protein content (15 µg) of sEVs was resolved in polyacrylamide gel electrophoresis and transferred into a polyvinylidene difluoride membrane. The membrane was blocked with 5% BSA in 1x Tris-buffered saline and was probed with CD9 (BioLegend), CD63 (Santa Cruz), NFkB (Cell Signaling Technologies), 20S proteasome subunit a3 (Santa Cruz Biotechnology), lipocalin (Abcam), TNFα and TGFβ (Cell Signaling Technologies), which were measured using specific antibodies. Binding was determined using secondary antibodies (anti-rabbit lipocalin, TNFα, TGFβ, and NFkB) and (anti-mouse 20S proteasome, CD63, and CD9) conjugated with horseradish peroxidase. The blots were washed with TBS Tween (Thermo Fisher Scientific), developed using chemiluminescent horseradish peroxidase substrate (Millipore Sigma) and exposed using the Odyssey CLx Imaging System (LICOR Biosciences). The band intensity of the target protein was quantified using ImageJ software and normalized with CD9 and CD63.

**Statistical analysis**

Student’s t test and paired t test were used as appropriate to compare the relative densities of sEVs isolated from the samples. Statistical analyses were carried out using Graphpad Prism.

**Results**

Compared to stable controls, patient 4 had a higher relative density of NFkB, 20S proteasome, lipocalin, TNFα, and TGFβ in sEVs isolated from plasma samples obtained 9 months before and 1 year after the onset of CLAD (P4B in Figure 3 in the manuscript). The differences in the relative density between two samples from patient two samples were not statistically significant.

**SUPPLEMENTAL FIGURE 1**

Histopathology images of pleural decortication specimen from patient 4 showing pleural fibrosis. A) Low power microscopy (hematoxylin and eosin stain, original magnification) showing areas of pleural fibrosis characterized by areas of hypocellular collagen deposition. B) High power microscopy (hematoxylin and eosin stain, original magnification x10) showing pleural fibrosis with organizing hemothorax/blood clot and minimal, predominantly chronic inflammation.

| **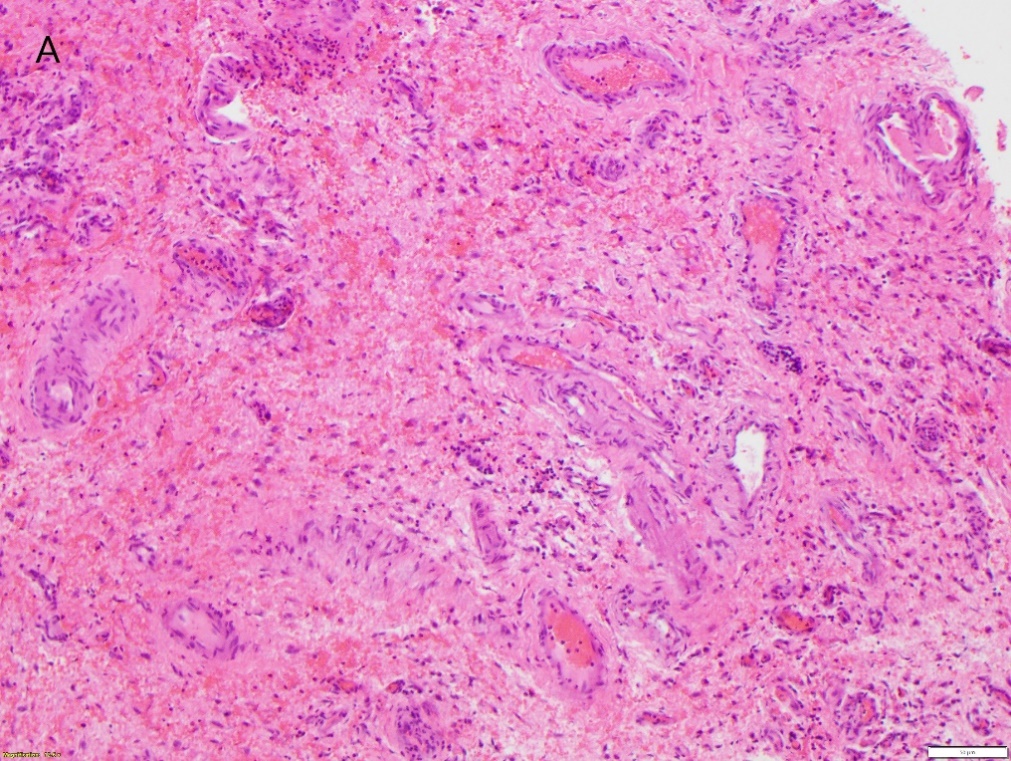** | 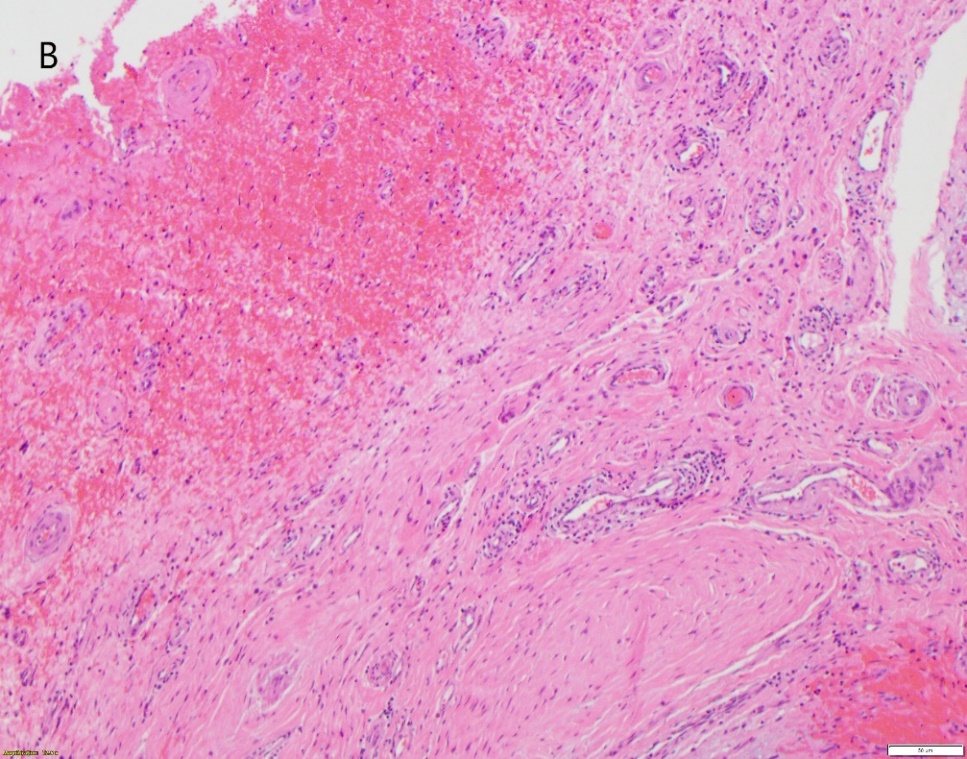 |
| --- | --- |

**SUPPLEMENTAL FIGURE 2**

**ELISA/western blot of small extracellular vesicles isolated from plasma from patient 4.** Red boxes indicate rows shown in Figure 3 of the manuscript.

**
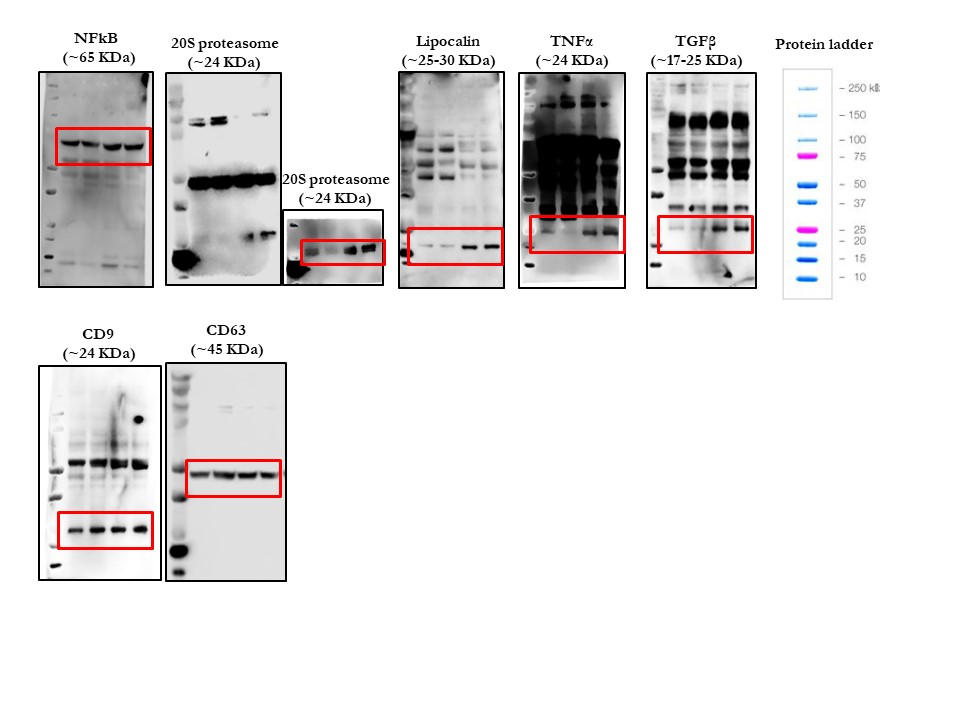
**
